# Algorithm for Automatic Forced Spirometry Quality Assessment: Technological Developments

**DOI:** 10.1371/journal.pone.0116238

**Published:** 2014-12-31

**Authors:** Umberto Melia, Felip Burgos, Montserrat Vallverdú, Filip Velickovski, Magí Lluch-Ariet, Josep Roca, Pere Caminal

**Affiliations:** 1 Dept. d'Enginyeria de Sistemes, Automàtica i Informàtica Industrial (ESAII), Centre for Biomedical Engineering Research (CREB), Universitat Politècnica de Catalunya, CIBER of Bioengineering, Biomaterials and Nanomedicine (CIBER-BBN), Barcelona, Spain; 2 Department of Pulmonary Medicine. Hospital Clínic de Barcelona (ICT). IDIBAPS, Universitat de Barcelona, Barcelona, Spain; 3 Centro de Investigación en Red de Enfermedades Respiratorias (CibeRes), Palma de Mallorca, Spain; 4 Barcelona Digital Technology Centre, Barcelona, Spain; 5 Dept. d′Enginyeria Telemàtica (ENTEL), Universitat Politècnica de Catalunya, Barcelona, Spain; 6 ViCOROB, Universitat de Girona, Girona, Spain; Technion - Israel Institute of Technology, Israel

## Abstract

We hypothesized that the implementation of automatic real-time assessment of quality of forced spirometry (FS) may significantly enhance the potential for extensive deployment of a FS program in the community. Recent studies have demonstrated that the application of quality criteria defined by the ATS/ERS (American Thoracic Society/European Respiratory Society) in commercially available equipment with automatic quality assessment can be markedly improved. To this end, an algorithm for assessing quality of FS automatically was reported. The current research describes the mathematical developments of the algorithm. An innovative analysis of the shape of the spirometric curve, adding 23 new metrics to the traditional 4 recommended by ATS/ERS, was done. The algorithm was created through a two-step iterative process including: (1) an initial version using the standard FS curves recommended by the ATS; and, (2) a refined version using curves from patients. In each of these steps the results were assessed against one expert's opinion. Finally, an independent set of FS curves from 291 patients was used for validation purposes. The novel mathematical approach to characterize the FS curves led to appropriate FS classification with high specificity (95%) and sensitivity (96%). The results constitute the basis for a successful transfer of FS testing to non-specialized professionals in the community.

## Introduction

Forced spirometry (FS) testing aims at a global assessment of lung and chest wall mechanics. Specifically, FS provides measurements of expiratory volume and flow during a maximal expiratory manoeuvre. It is a relevant test in the clinical setting useful to perform both diagnosis and assessment of functional reserve in various lung-related health disorders. The test is also used for pre-operative evaluation and assessment of disability/impairment. Moreover, there is evidence that key spirometric indices (FVC, forced vital capacity; and, FEV1, forced expiratory volume during the first second) predict survival in the general population. For all these reasons, it is forecasted that the role of FS testing will expand across healthcare tiers and beyond respiratory medicine.

As part of the FS testing procedure, the patient performs maximum expiratory maneuvers under the guidance of a healthcare professional who should: (1) aim for a proper cooperation of the patient; (2) assess the quality of different FS manoeuvres; and, (3) select the most suitable spirometric values using the ATS/ERS recommendations [1].

The equipment for FS measurements and the recommendations for testing are highly standardized [2], [3], as well as the quality assessment [1]. The current systems measure expired flow using different technologies [4] that generate two types of FS curves: (1) a volume-time curve (VT) representing volume (in liters, L) along the ordinate and time (in seconds, s) in the abscissa; and, (2) a flow-volume curve (FV) depicting expired flow (in liters per second) in the ordinate and expired volume (in liters) in the abscissa. Clinically useful spirometric indices (i.e FVC and FEV_1_) are calculated from selected curves following the international recommendations [1]–[3]. FS testing requires a high degree of patient cooperation with the support of a health professional to ensure that the quality of the maneuvers follows the recommended standards [1]. The transfer of FS testing to non-specialized professionals in the community generates a challenge in terms of preserving the quality of the testing to preclude misdiagnosis due to poor quality of FS curves.

There is evidence that remote off-line support of quality testing shows both feasibility and cost-effectiveness, but requires supervision by a specialist [5]. Unfortunately, currently available equipment with functionalities for automatic assessment of quality of FS testing generates poor outcomes due to an inadequate application of the ATS/ERS recommendations on quality control [5], [6]. We recently reported the high potential of an automatic algorithm for real-time assessment of quality testing paving the way for the transfer of FS testing to the community [5], [7].

It is hypothesized that the regional deployment of a comprehensive program ensuring: *i)* reliable automatic quality assessment of forced spirometric testing; ii) off-line remote assistance to non-specialized health professionals; and, *iii)* accessibility to quality labeled forced spirometric information across healthcare tiers, may have a marked positive impact on quality of diagnosis, healthcare outcomes and may generate cost savings.

The current research reports the identification of new metrics based on a mathematical approach that describes the entire spirometric curve allowing a proper quality assessment of volume-time (VT); flow-time (FT); and, flow-volume (FV) curves. It is of note that the results are under review in the European Patent Office with the registration number (PCT/EP2013/068732).

## Materials and Methods

### Databases

Three databases were used for building and validating the algorithm: (1) 24 simulated curves recommended by the ATS [2], [3]; (2) 270 curves from 90 patients examined at the Hospital Clinic de Barcelona [5] (P1); and, (3) 778 curves from 291 patients (P2) from one of the Primary Care centers in Barcelona. Forced spirometry testing in P1 and P2 was performed with the same equipment (Sibel 120, SIBELMED, Barcelona Spain). The simulated curves permitted the elaboration of the initial version of the algorithm; whereas the two patient databases (P1 and P2) were considered for refinement and validation purposes, respectively. The study was approved by the Ethical Committee of the Hospital Clínic de Barcelona. All the participants signed informed consent.

### Algorithm development

The 24 simulated ATS curves were used to perform a comprehensive characterization of the curve morphology to facilitate the application of the different quality criteria defined in the ATS/ERS recommendations [2], [3]. To this end, three different concepts were introduced as defined below:

#### Criterion

Specific feature of the spirometric testing that requires quality assessment (i.e. back extrapolation, end-of-curve, peak flow, etc…). The quality analysis of the different criteria considered by the algorithm will provide an overall quality assessment of the spirometric curve.

#### Metric

Mathematical description of a given criterion. Several criteria may require one or more metrics to be properly defined.

#### Threshold

Quantitative values of a given metric used to assess the quality of a criterion. It is of note that some metrics may have primary and secondary thresholds.

The ultimate aim of the algorithm was to integrate the new criteria to enhance current quality assessment [1] and to allow on-line quality control of testing.

Each criterion (*C_n_*) defined with the 24 ATS curves used one (primary) or more (secondary) metrics (*M_j_*) with the respective threshold(s). In each step of the algorithm development, the results were compared with the criteria of one expert in the field of pulmonary function testing.

**Figure 1:**
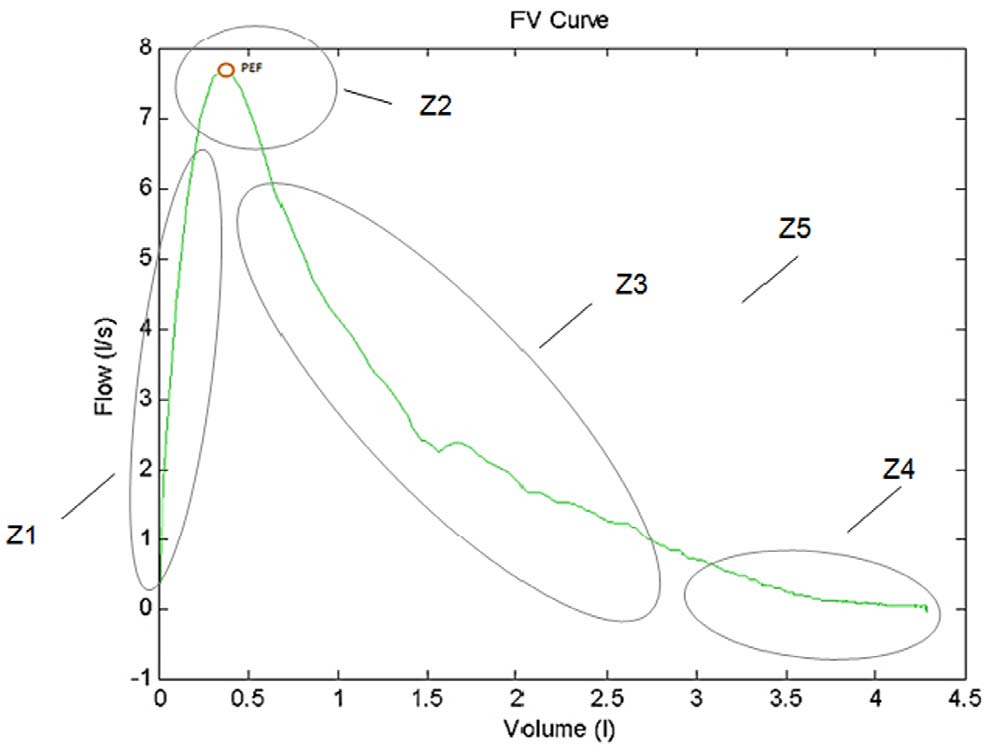
Spirometer Zones. An example of FV curve with five zones described.

In order the perform a global assessment of each spirometric curve, five different zones were identified in the flow-volume graph, as indicated in Fig. 1 and described in detail in S1 File. The overall result of applying the new methodology was the identification of three different quality grades, namely: i) Grade 0 → curve to be rejected because of a bad morphology; ii) Grade 1 → curve with acceptable morphology; and, iii) Grade 2 → curve requiring specialized professional judgment for acceptability.

The refinement of the threshold values was performed in an iterative process to maximize the agreement between the human expert and the automatic classification in grades 0 and 1 and to minimize the number of curves automatically classified in grade 2. The first two categories, grades 0 and 1 allow proper real-time and automatic classification of FS; whereas grade 2 requires off-line expert assessment. The automatic grade assignment is made as described in the S1 File.

The algorithm issued from the analysis of the 24 ATS curves was subsequently evaluated using the P1 database following identical procedures.

**Figure 2:**
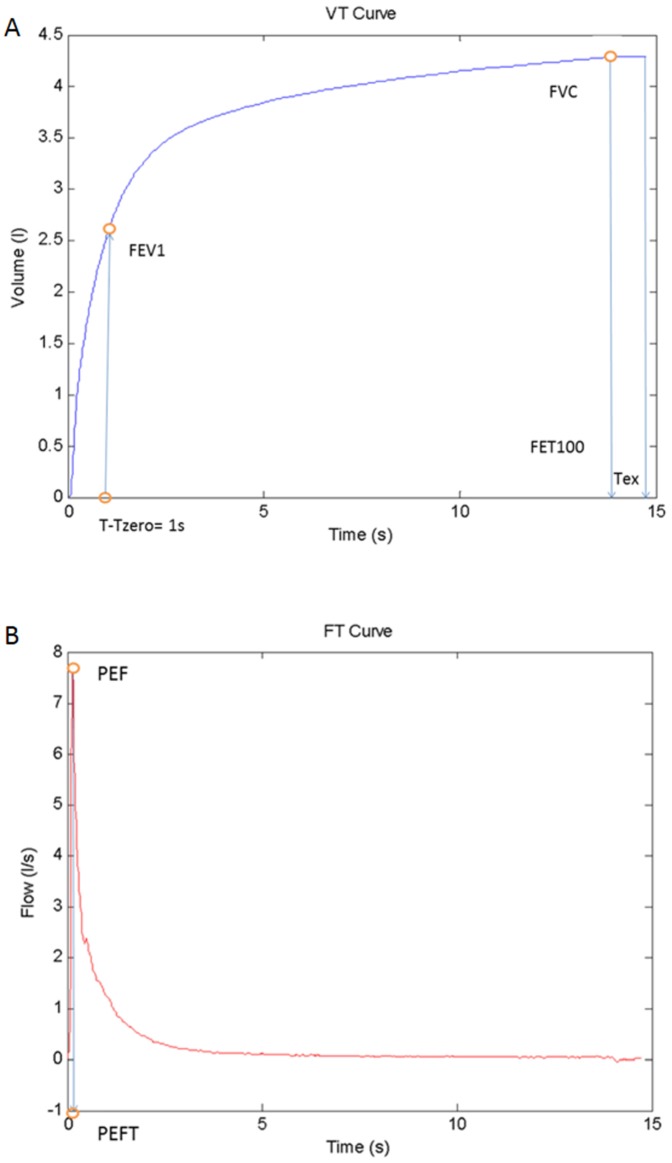
Spirometer metrics. Metrics involved in the traditional criteria: (A) *FVC* and *FEV1*, (B) *PEFT*.

The current algorithm incorporates the 4 traditional ATS/ERS criteria commonly used in commercially available equipment with automatic quality assessment and applies several other ATS/ERS criteria for quality control of FS as indicated below in the description of the corresponding zones and in Fig. 2. The metrics corresponding to the 4 traditionally used ATS/ERS recommendations in commercially available equipment are defined as follows:

- *BEV* refers to back extrapolated volume (BEV>0.15 L or BEV <5% of FVC), which is the volume value for t = *T_zer_* (*T_zero_* refers to the back extrapolated time, which is the time at which the volume curve tangent with maximum slope crosses the horizontal time axis).
- *EOTV* refers to the difference between maximum and minimum volume in the last 1 second of exhalation. (*T_ex_* refers to the time from *T_zero_* to the time in which the VT curve reaches *EOTV* <0.025 L or the end of exhalation, as depicted in Fig. 1A),
- *FET100* refers to the time from *T_zero_* to the time in which the VT curve reaches *FVC*, as depicted in Fig. 1A (6 seconds in adult population).
- Repeatability criteria (three good maneuvers, two of them with differences in FVC and FEV_1_ less than 0.15 L).

### Five spirometric zones

Fig. 2 displays the rationale for the five spirometric zones considered in the current analysis. The first zone (Z1) encompasses the area from zero to peak flow; whereas Z2 relates to the profile or the peak expiratory flow (PEF). The decrease of flow rate after the peak is analyzed in Z3; the end-of-test area is examined in Z4 and, finally, Z5 considers the overall shape of the curve.

**Figure 3:**
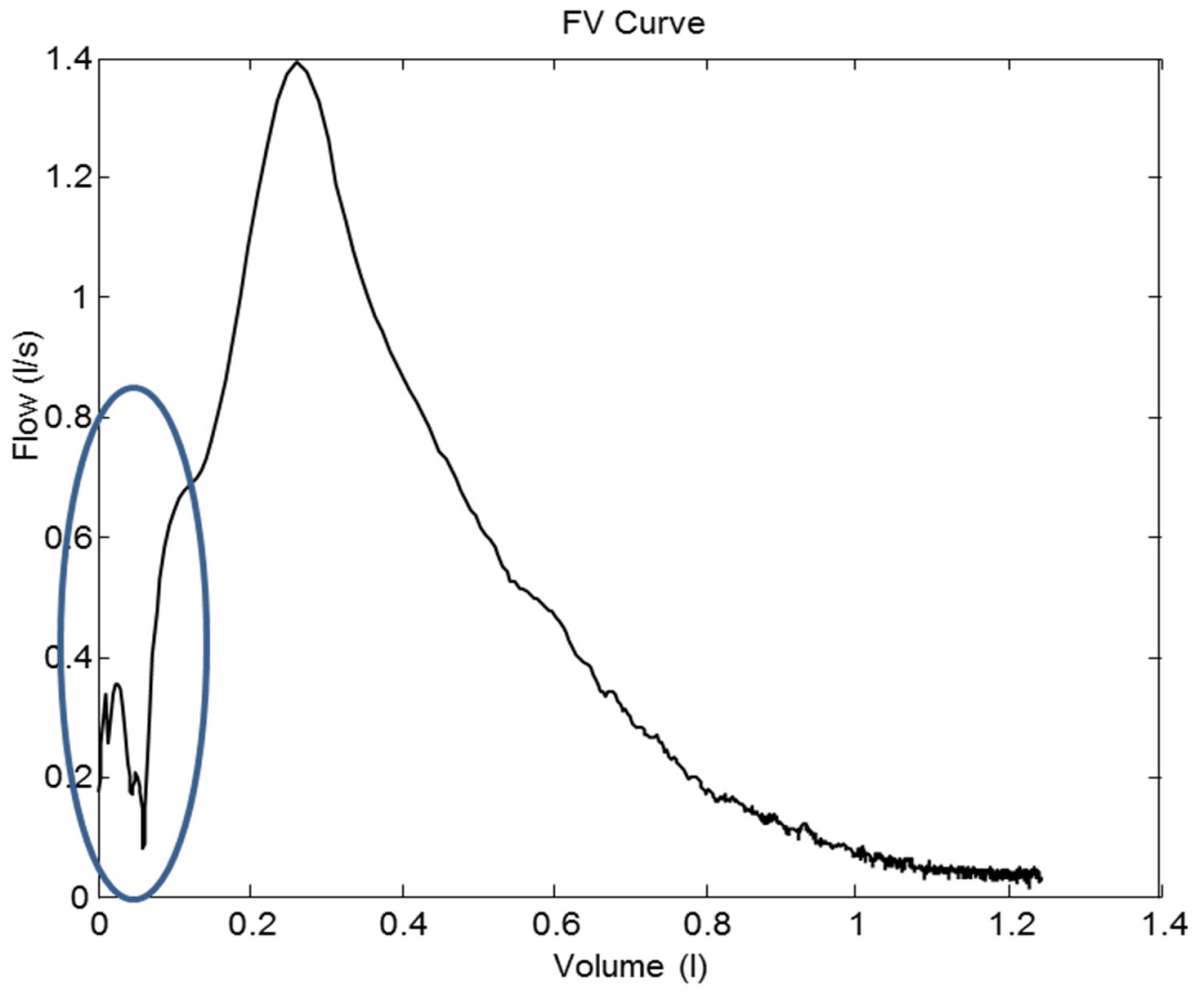
Zone Z1 analysis. An example of a FV curve that presents irregularity on the ascent to the PEF.

The Z1 criteria ensure that the slope of the curve is regular and free from fluctuations. The calculations are based on the first and second derivatives of the FV curve in zone Z1. Fig. 3 depicts an example of an FV curve that presents a bad morphology in zone Z1. The criterion *C_1_* detects that irregular concavity or convexity exists. A second criterion *C_2a_* detects that the profile has an irregular slope. The criterion *C_2b_* detects that the profile has an irregular concavity or convexity.

**Figure 4:**
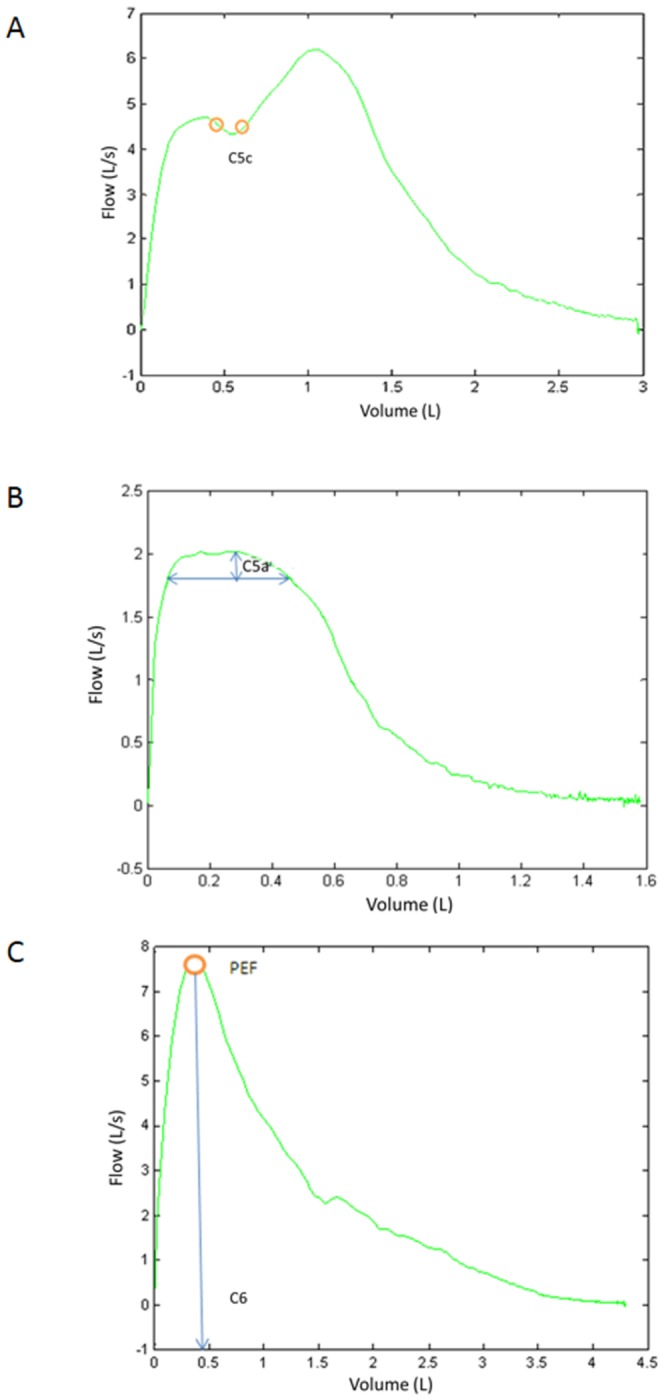
Zone Z2 analysis. Examples of FV curves that present (A) bimodal peak; (B) flat peak and (C) slow peak.

The Z2 criteria ensure that the PEF occurs at an early point in the maneuver, it has an appropriate height to width ratio and there are no secondary peaks present. Fig. 4 depicts example curves in which the corresponding tests are performed in the zone Z2. Criterion *C_3_*, detects that the PEF point has occurred too late. Criterion *C_4_* detects that the PEF point is too early. The criteria *C_5_* analyze the peak. *C_5a_* and *C_5b_* detect a flat peak (Fig. 4B). The criterion *C_5c_* detects a situation of bimodal peaks as depicted in Fig. 4A (multiple peaks). The criterion *C_6_* detects if the V value in the position of the PEF is lower than a fixed threshold (Fig. 4C).

**Figure 5:**
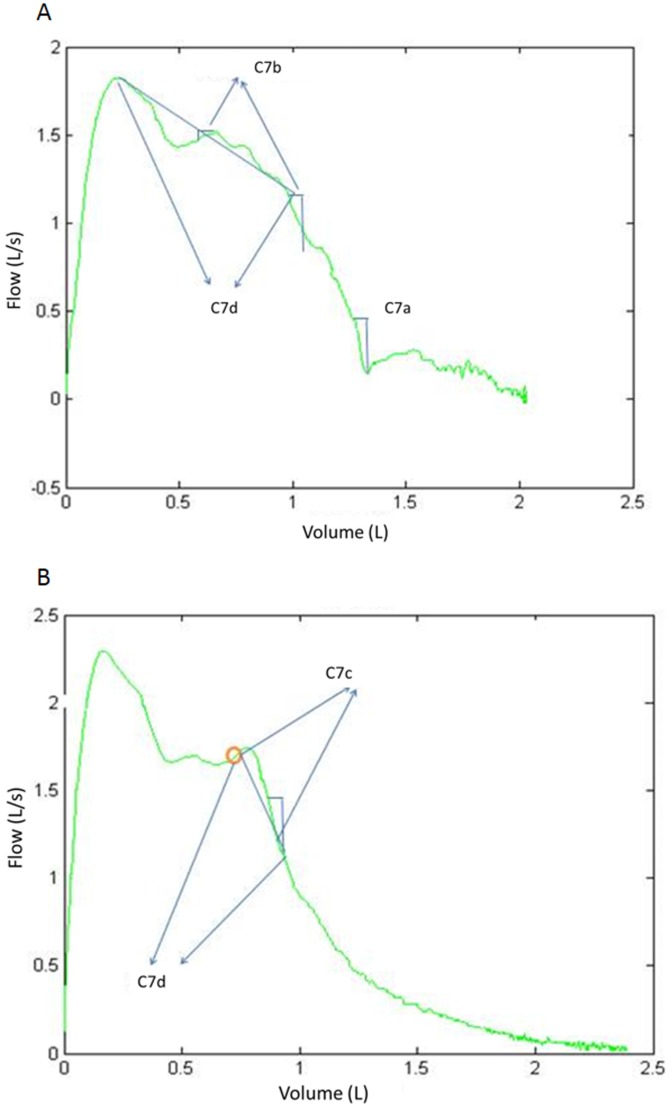
Zone Z3 analysis. Examples of FV curves that present irregularity in the descent from PEF.

The Z3 criteria ensure that the slope of the curve is regular and free from fluctuations and are based on the first derivative, and definite integrals of the FV curve in zone Z3. Fig. 5 depicts an example curve in which the corresponding tests are performed in zone Z3. Fig. 5A depicts criterions *C_7a_*, *C_7b_* and *C_7d_*. Fig. 5B depicts criterions *C_7c_* and *C_7d_*. The criterion *C_7a_* detects a situation of high slopes during FV curve descent. The criterion *C_7b_* detects an excessive variation in the slope of the FV curve in zone Z3. The criterion *C_7c_* detects an excessive variation in the slope calculated in a V segment of the FV curve in the zone Z3. The criterion *C_7d_* detects an irregular slope.

**Figure 6:**
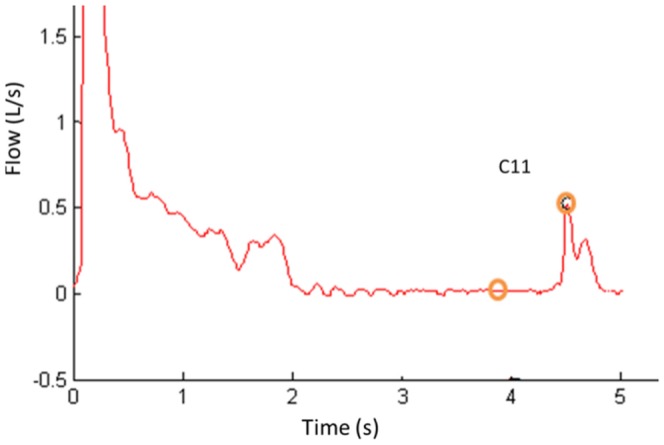
Zone Z4 analysis. Example of FT curve that present irregularity in the final part.

The Z4 criteria ensure that the flow at the end of the curve is regular and free from fluctuations and are based on the first order derivative of the FT curve in zone Z4, and the difference between the maximum and minimum volume in the last second of exhalation. Fig. 6 depicts an example curve in which the corresponding tests are performed in the zone Z4. Fig. 6A and 6B depict criterion *C_11_*. Criteria *C_8_* is the traditional *BEV* criteria (*BEV*>0.15 L or *BEV* <5% of *FVC*). Criteria *C_9_* is the traditional *EOTV* criteria (*V*<0.025 L in *t*≥1 s). Criteria *C_10_* is a combination of 5 criteria explained in the following lines. *C_10a_* detects if the *EOTV* and *Tex* both does not satisfy their traditional criteria. *C_10b_* defines a new period to calculate *EOTV* if the *Tex* traditional criteria is satisfied. *C_10c_* defines a new threshold for *EOTV* if the *Tex* traditional criterion is satisfied. *C_10d_* uses threshold to define the *EOTV(Tex)* and compare with the traditional threshold. *C_10e_* defines *EOTV* calculated as a function of *Tex*. Criterion *C_11_* detects irregularity or oscillation at the end part of FT curve.

**Figure 7:**
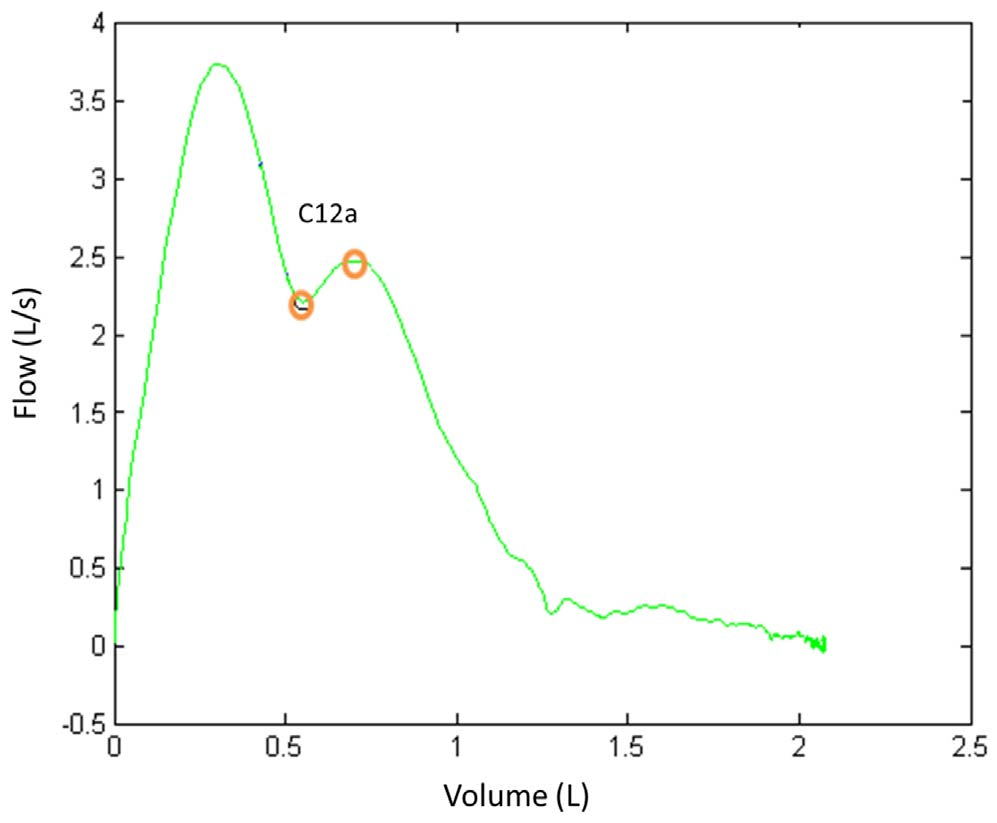
Zone Z5 analysis. Examples of FV curve with peak and valley.

The Z5 criteria ensure that there only exists one local maximum (the *PEF* point) and they are based on the derivative of the FV curve. Fig. 7 depicts criterions *C_12a_* and *C_12b_*. The criterion *C_12a_* detects a situation of multiple peaks that typically occurs when the subject coughs. The criterion *C_12b_* detects a situation of multiple peaks for values of *V* adjacent to *FEV_1_*.

For more details, see S1 File.

### Algorithm evaluation

The quality grades (0 to 2) generated by the algorithm using the P2 dataset were compared with those generated by the expert evaluator. Sensitivity and specificity of the algorithm were calculated for all curves classified as classes 0 or 1 in order to quantify the agreement between the algorithm and the evaluator and between the 4 traditional ATS criteria and the evaluator. Sensitivity is defined as the number of curves classified as class 0 by both the algorithm (or the 4 traditional ATS/ERS criteria) and the evaluator divided by the total of the curve evaluated in grade 0 by the evaluator, while specificity is defined as the number of curves classified as class 1 by both the algorithm (or the 4 traditional ATS/ERS criteria) and the evaluator divided by the total of the curve evaluated in grade 1 by the evaluator.

## Results and Discussion

**Table 1** summarizes the evaluation of the new algorithm showing that a reasonable percentage of FS curves (88%), could be automatically assessed as either acceptable (grade 1) or unacceptable (grade 0) in concordance to an expert evaluation. Twelve percent of the curves (n = 93) were automatically classified as grade 2 requiring an expert opinion. It is of note that 43% of these grade 2 curves were evaluated as grade 0 and 57% as grade 1 by the expert evaluator. The table also compares the results of the current research against those obtained only using the four traditional ATS/ERS criteria for quality assessment. Think

**Table 1:** Computed sensitivity (Sen) and specificity (Spe) using the current automatic classification algorithm and using only the four traditional ATS/ERS quality criteria applied to P2.

Automatic Classification Algorithm	Sen: 96.1%
	Spe: 94.9%
*Number of Curves Detected in each Grade*	*Grade 0*: 266
	*Grade 1*: 419
	*Grade 2*: 93

Four traditional ATS/ERS Criteria	Sen: 67.7%
	Spe: 75.0%
*Number of Curves Detected in each Grade*	*Grade 0*: 320
	*Grade 1*: 458
*TOTAL CURVES ANALYZED*: 778	

Several alternative technological approaches [8]–[14] for automatic quality assessment of FS testing were considered during the current study design. But, we consider that the method reported allows a comprehensive and efficient application of the different quality control ATS/ERS criteria [1], it does not show limitations in terms of computer requirements, or unnecessary delays using regular computers used in the clinical setting, and it was well accepted by health professionals as on-line clinical decision support systems support during performance of FS testing.

We acknowledge as a limitation of the current study that the algorithm has been developed with the feedback of only one expert. Consequently, despite the positive results reported, there is a need for a formal assessment of the variability among various expert observers. Despite that internal interim data indicates that interobserver variability is not a relevant factor, we are planning its evaluation as part of a large prospective future trial analyzing both clinical and cost saving impact of the regional deployment of the high quality FS program, as described below.

The need for an external, likely centralized, quality control of FS testing [15]–[16] is widely accepted if based on well-established objective criteria. It is of note, however, that low specificity of any combination of the computer-based quality control criteria using only the four traditional ATS/ERS [3] has been reported [5], [6] such that automatic quality assessment using algorithms incorporated in commercially available equipment cannot replace the visual inspection by an expert. In contrast, our proposed algorithm shows two advantages: it enhances quality control of FS testing and allows on-line assessment of the testing.

Previous reports have indicated the potential of telemedicine to enhance both quality and diagnostic potential of FS testing carried out by non-expert professionals [5], [17]–[19], but the studies are based on off-line analyses by specialists [20]–[22]. The findings of the current research suggest that a vast majority of FS testing carried out by non-specialized professionals in primary care could be reliably assessed in real-time. Consequently, the results of the current study refine previous achievements [5] and open the way to explore extensive and efficient adoption of this type of high quality FS programs.

Several factors limiting regional deployment of a quality control program of FS using the current algorithm in the clinical practice are acknowledged, namely: (i) implementation of standardized raw spirometric data transfer through a clinical document architecture (CDA) [23]; (ii) an ICT architecture providing interoperability across healthcare tiers; (iii) design of an educational program for professionals; and, (iv) implementation of incentives fostering professional engagement. The region of Catalonia will be ready in 2015 for the regional deployment of a high quality FS program overcoming the barriers alluded to above. Such a comprehensive program: *i)* will likely have a positive clinical impact on the quality of diagnosis of patients with respiratory disorders, *ii)* should prevent unnecessary duplication of FS testing; *iii)* will likely enhance longitudinal follow-up of patients and support cost-effective preventive strategies aiming at modulating disease progress; *iv)* will pave the way to generate novel approaches to assess abnormal biological variability of FS testing; and, *v)* may likely produce cost savings. No doubt that such a program will require a proper evaluation on a longitudinal basis to assess its potential for generation of healthcare value.

## Conclusion

The results of the current study provides a tool that makes operational a comprehensive application of the ATS/ERS recommendations for automatic quality control of FS testing. It constitutes a pivotal element facilitating the design and future deployment of a high quality FS program based on remote automatic evaluation of the testing.

## Supporting Information

S1 File **Metrics and flow-chart.** This file provides a detailed description of the metrics and the decision process used in each of the zone (Z1, Z2, Z3, Z4 and Z5) in order to automatically evaluate the FS curves.
(PDF)
