# Supplementary material for: Algorithm for Automatic Forced Spirometry Quality Assessment: Technological Developments
Source: PLoS One. 2014 Dec 31;9(12):e116238. doi: 10.1371/journal.pone.0116238 (PMC4281176; doi:10.1371/journal.pone.0116238)
Supplement: S1 File — Metrics and flow-chart. This file provides a detailed description of the metrics and the decision process used in each of the zone (Z1, Z2, Z3, Z4 and Z5) in order to automatically evaluate the FS curves. (PDF) [file pone.0116238.s001.pdf]

Algorithm for Automatic Forced Spirometry Quality Assessment: Technological Developments

Umberto Melia[1], Felip Burgos[2-3], Montserrat Vallverdú[1], Filip Velickovski[4], Magí Lluch-Ariet[4-5], Josep Roca[2-3], Pere Caminal[1]

[1] Dept. ESAII, Centre for Biomedical Engineering Research, Universitat Politècnica de Catalunya, CIBER of Bioengineering, Biomaterials and Nanomedicine (CIBER-BBN), Barcelona, Spain.
[2] Department of Pulmonary Medicine. Hospital Clínic de Barcelona (ICT). IDIBAPS. Universitat de Barcelona, Barcelona.
[3] Centro de Investigación en Red de Enfermedades Respiratorias (CibeRes), Palma de Mallorca.
[4] Barcelona Digital Technology Centre
[5] Dept. d'Enginyeria Telemàtica (ENTEL), Universitat Politècnica de Catalunya, Barcelona

Correspondence: Umberto Melia. Centre de Recerca en Enginyeria Biomèdica de la Universitat Politècnica de Catalunya (CREB-UPC), Pau Gargallo 5, 08028 Barcelona. Spain. E-mail: umberto.melia@upc.edu. Phone: + 34 93 401 71 60.

On-Line Supplement

Based on the comparison of the metric (M_j) with its related threshold (STh_i), a score value (S_n) is assigned to each criterion (C_n). Then, for each S_n a score threshold (STh_n) is defined in order to evaluate the quality of the criteria. The score of some criteria are jointly analyzed by summing their scores to be compared with a single score threshold. The automatic classification of the FS curves is determined by considering each score (or combination of some scores) and the respective score thresholds, as it is shown in the flow-chart of each zone.

Scores

Each score (S_n) is calculated as function of the metrics (M_n) of each criterion and the respective thresholds. When the criterion C_n condition is $M_n > th_{ni}$, S_n= 100 if $M_n > th_{ni}$ otherwise S_n=100$\frac{M_n}{th_{ni}}$. When the criterion C_n condition is $M_n < th_{ni}$, S_n= 100 if $M_n < th_{ni}$ otherwise S_n=100100$\frac{th_{ni}}{M_n}$

If a particular criterion uses more than one metric for defining its quality, the score of the criterion is calculated as the mean value of the scores of each metric.

Flowchart

Figure S1, S2, S3, S4 and S5 show the flowchart of the decision process of the entire algorithm, divided by zone.

Each block is assessed by a score (S_n) or a combination of scores compared with a score threshold (Sth_n). On the left of the flowchart there is the main path followed by the curves that fulfill all the criteria. If all the criteria scores are above of respective thresholds a curve remains

in the main path and finally it is evaluated as grade 1. If a curve does not fulfill a criterion score it follows the arrow on the right where there are two different path options depending on the block:

- The curve follows the arrow on the right and finally is evaluated as grade 0
- The curve enters in one or more control boxes and if the score is above the threshold, the curve is evaluated as grade 2 and returns to the main path; otherwise the curve follows the arrow on the right and finally is evaluated as grade 0.

Each score (S_n) refers to the respective criterion (C_n) except the S_{13} (TOT SCORE) that is calculated as the sum of all the S_n of all the criteria (n): $S_{13} = \sum_n S_n$ and it is used in the final control.

Zone Z1 Analysis

The zone starts at the beginning of the spirometry test and ends once it has been determined that the peak exhalation flow PEF has occurred. The criteria ensure that the slope of the curve is regular and free from fluctuations and are based on the first and second derivative of the FV curve in the zone Z1. Table S1 lists the different threshold used for the comparison tests of the metrics.

The metric M_1 (*Diff2*) is defined as the second order derivative of the FV curve in Z1. The metric M_2 (*AreaDiff*) is defined as the sum of the negative values of the first order derivative of the curve. The metric M_3 (*AreaDiff2*) is defined as the sum of the positive values of the second order derivative of the FV curve.

A set of criteria have been developed to detect irregular concavity or convexity before the PEF value. In the criterion C_1 (*Diff_single*) it is determined that irregular concavity or convexity exists if the extracted signal metric has a second order derivative which exceeds the threshold of metric M_1. In a second criterion C_{2a} (*Area Diff*) it is determined that the profile has an irregular slope if M_2 exceeds the 50 % of the sum of derivative values of zone Z1. In the criterion C_{2b} (*Area Diff2*) it is determined that the profile has an irregular concavity or convexity if the sum of the threshold of metric M_3 exceeds the sum of all derivative values of the zone Z1. Figure S1 depicts the flow-chart of the decision process in Z1.

TABLE S1

All the Z1criteria associated with metrics and respective thresholds

N	**CRITERION (C_n)**	**METRICS**	**MAIN THRESHOLD (th_{ni}) $i=1$**	**VALUE**
**C1**	**Diff_single**	M_1	*diff2Th*	0.1 L/s^2
**C2a**	**Area Diff**	M_2	*diffAreaTh*	50 % of the sum of all derivative values of the interval
**C2b**	**Area Diff2**	M_3	*diff2AreaTh*	100 % of the sum of all derivative values of the interval

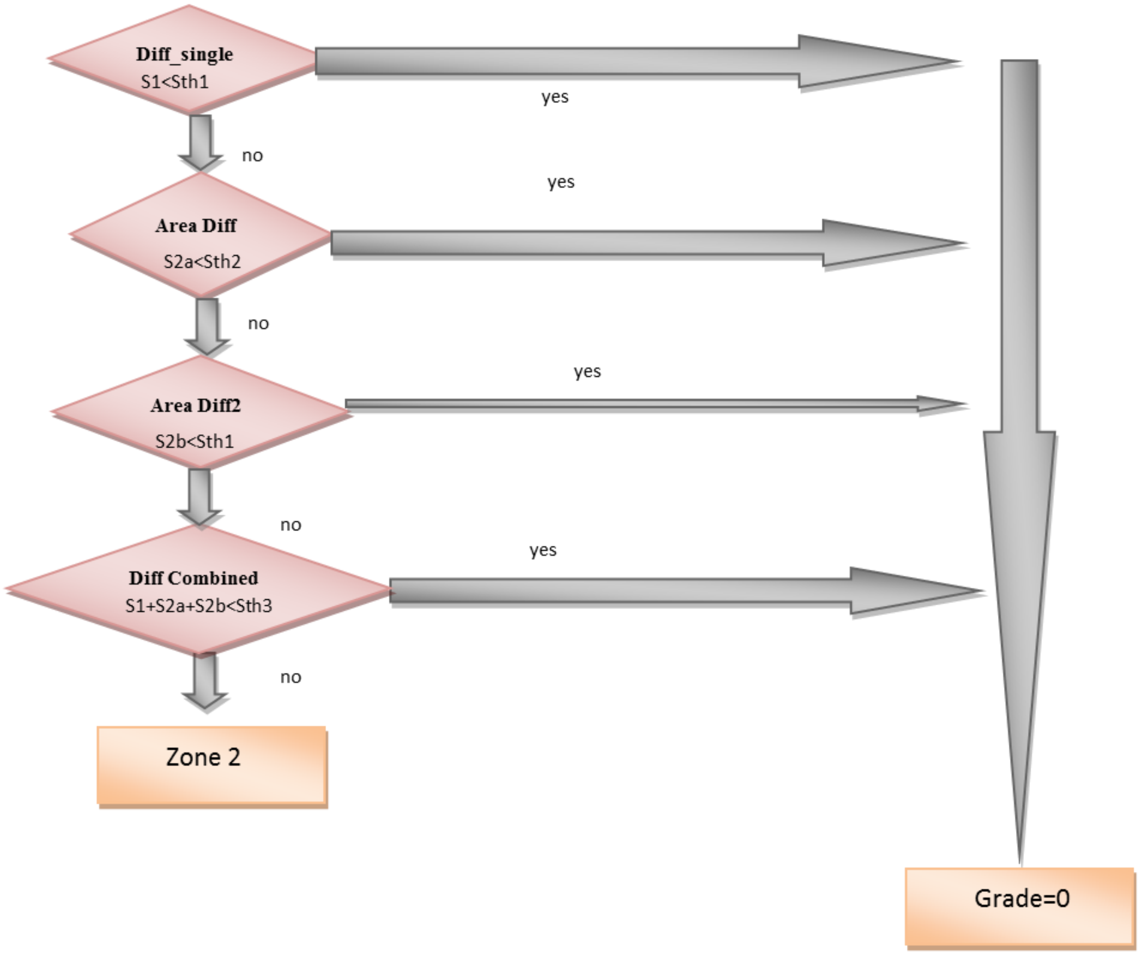

Figure S1 – Flowchart of the algorithm in Z1: in each decision box the curve scores (*Sn*) or the combination of scores are compared with the respective threshold (*Sthn*), the rectangular box are used to assign a grade to the curve or to go to the following zone.

Zone Z2 Analysis

This group of criteria has been developed to analyze the morphology of the maneuver around the *PEF* point in *VT*, *FT*, and *FV* profiles. The zone is centered about the PEF point. The criteria ensure that the PEF occurs at an early point in the maneuver, it has an appropriate height to width ratio and there are no secondary peaks present. Table S2 lists the different threshold used for the comparison tests of the metrics in each criterion.

The metrics M_4 and M_5 are based on the intersection between the FV curve and a horizontal line, the height position of the horizontal line is M_4, while the length is M_5. These metrics ensure that PEF must be a peak in FV curve. Threshold of the peak is fixed relative to FVC (*PEF Peak*) or relative to FEV1 (*PEF Peak2*). The metric M_6 (*PEFDouble*) aims to detect a “PEF bimodal” and corresponds to the difference between absolute maximum and consecutive relative maximum of FV curve. The metric M_7 is defined as the corresponding volume value at the PEF point .Traditional metrics *FEV1*, *FVC* and *PEFT* are also used. In criterion C_3, it is determined that PEF point occurs too late if the corresponding time at the PEF occurrence is greater than a set threshold (*ThPEFup*). In criterion C_4 it is determined that the PEF point is too early if the corresponding time at which the PEF occurs is less than a set threshold (*ThPEFdown*). The criterion C_5 analyzes the peak. C_{5a} and C_{5b} detect a flat peak. The criterion

C_{5c} detects a situation of bimodal peaks (multiple peaks) when a secondary local maximum in the FV curve has *F* value higher than a fixed threshold percentage (*ThBIPEF*) of PEF. Figure S2 depicts the flow-chart of the decision process in Z2.

TABLE S2

All the Z2 criteria associated with metrics and respective thresholds

N	CRITERION (C_n)	METRICS	MAIN THRESHOLD (th_{ni}) $i=1$	VALUE	SECONDARY THRESHOLD (th_{ni}) $i=\{2,3\}$	VALUE
**C3**	**PEF Time Up**	PEFT	*ThPEFup*	0.12 s		
**C4**	**PEFTimeDown**	PEFT	*ThPEFdown*	0.25 ms		
**C5a**	**PEF Peak1**	M_4 and M_5	*ThOriz1*	15 % of FVC	*ThVert1*	10 of % PEF
**C5b**	**PEF Peak2**	M_4 and M_5	*ThOriz2*	17.5 % of FEV1	*ThVert2*	12.5 % of PEF
**C5c**	**PEFDoubleTest**	M_6	*ThBIPEF*	30 % of PEF		
**C6**	**PEF Slow test**	M_7	*ThPefSlow*	20 % of FVC		

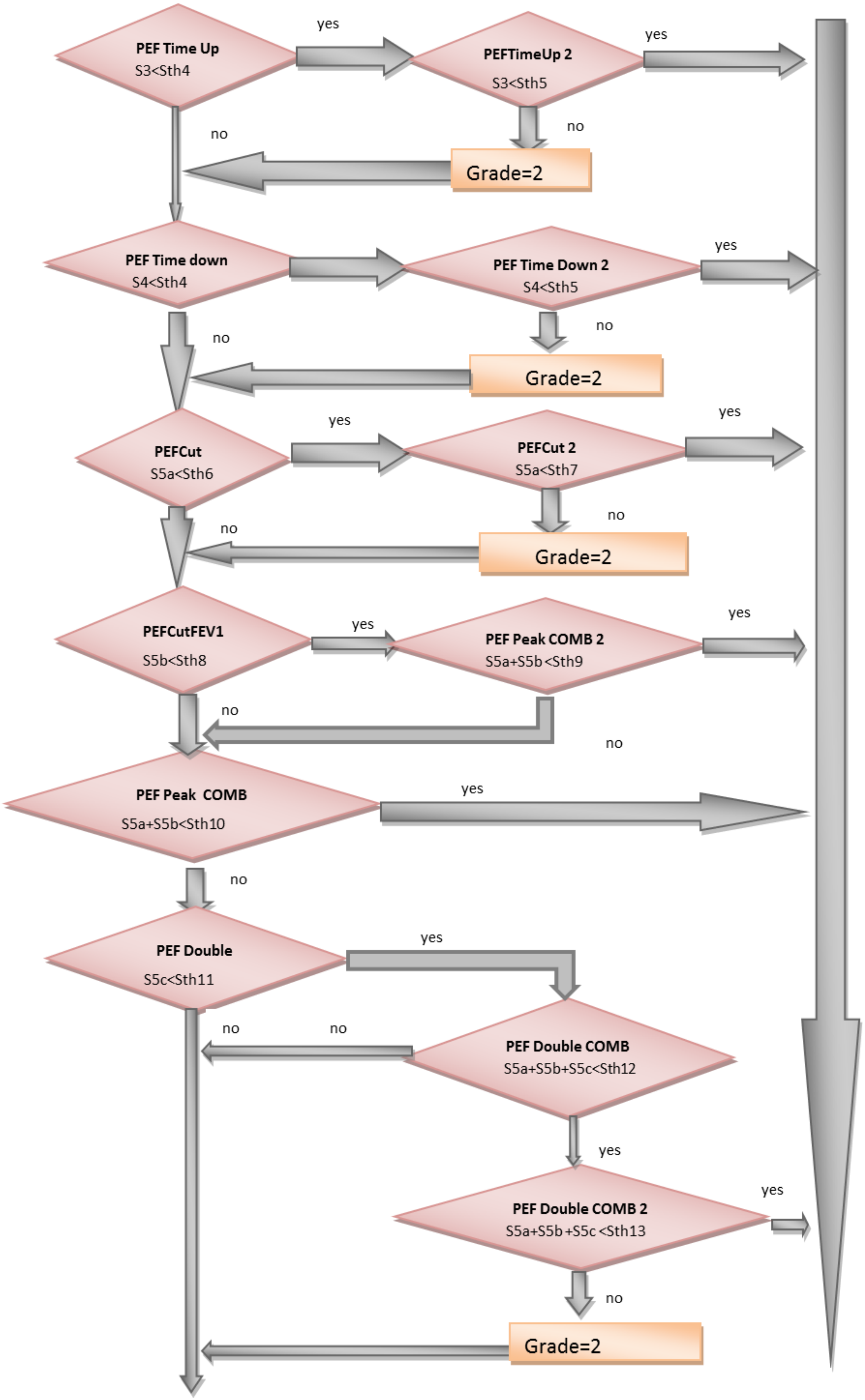
PEF Time Up
S3<Sth4
yes
PEFTimeUp 2
S3<Sth5
yes
no
no
Grade=2
PEF Time down
S4<Sth4
PEF Time Down 2
S4<Sth5
yes
no
no
Grade=2
PEFCut
S5a<Sth6
yes
PEFCut 2
S5a<Sth7
yes
no
no
Grade=2
PEFCutFEV1
S5b<Sth8
yes
PEF Peak COMB 2
S5a+S5b<Sth9
yes
no
no
PEF Peak COMB
S5a+S5b<Sth10
yes
no
PEF Double
S5c<Sth11
yes
no
no
PEF Double COMB
S5a+S5b+S5c<Sth12
yes
PEF Double COMB 2
S5a+S5b+S5c<Sth13
yes
no
Grade=2

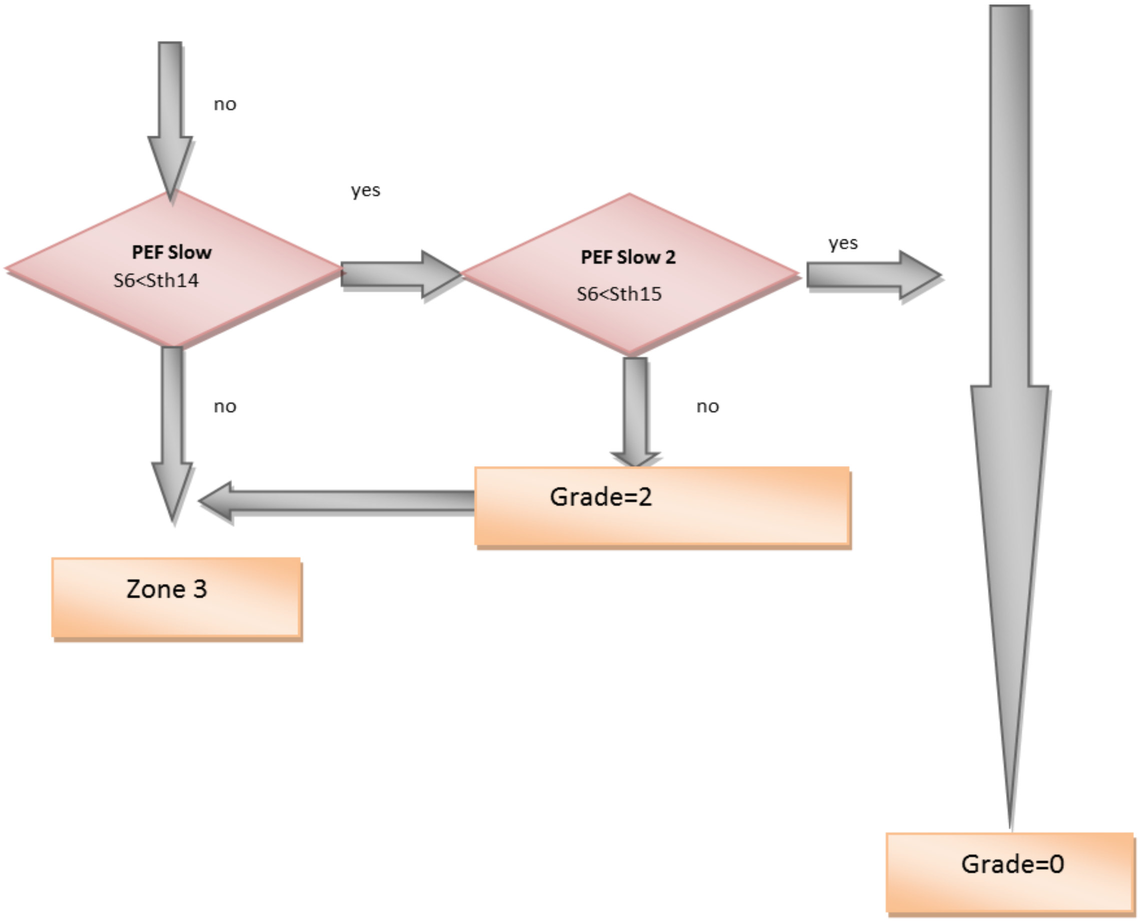

Figure S2 – Flowchart of the algorithm in Z2: in each decision box the curve scores (*Sn*) or the combination of scores are compared with the respective threshold (*Sthn*), the rectangular box are used to assign a grade to the curve or to go to the following zone.

Zone Z3 Analysis

This group of criteria has been developed to analyze the descent in the FV curve. The zone starts at the beginning of the PEF point and ends before the end of the FV curve. The criteria ensure that the slope of the curve is regular and free from fluctuations and are based on the first derivative, and definite integrals of the FV curve in the zone Z3. Table S3 lists the different threshold used for the comparison tests of the metrics.

The metric M_8 is defined as the first order derivative of the FV curve in Z3. The metric M_9 is defined as the area between FV curve and a line from the PEF to the point in which the magnitude of the first order derivative exceeds a predefined threshold. Traditional metrics *PEF*, *FEV1* and *FVC* are also used.

The criterion C_{7a} detects a situation of high slopes during FV curve descent when the maximum value of the first order derivative of the FV curve exceeds a threshold percentage (*Max_Slope*) of PEF for volume values above a threshold percentage (*PosFVSI*) of FVC. The criterion C_{7b} detects an excessive variation in the slope of the FV curve in the zone Z3 when the first order derivative exceeds a threshold (*MaxSlope* / *KSlopeTest*) for subsequent values of *V* that occur after the point at which the first order derivative falls below a threshold percentage (*Min_Slope*) of PEF. The criterion C_{7c} detects an excessive variation in the slope calculated in a *V* segment of length *ThSlopeCalc* of the FV curve in the zone Z3 when the first order derivative exceed a threshold (*ThDerivTest*) for subsequent values of *V* it cannot go up the threshold *ThSlTest2* % of PEF. The criterion C_{7d} detects an irregular slope by combining the previous M_8

and M_9. Figure S3 depicts the flow-chart of the decision process in Z3.

TABLE S3

All the Z3 criteria associated with metrics and respective thresholds

n	CRITERION (C_n)	METRICS	MAIN THRESHOLD (th_{ni}) $i=1$	VALUE	SECONDARY THRESHOLD (th_{ni}) $i=\{2,3\}$	VALUE
**C7a**	**FV_Max_Slope**	M_8	*Max_Slope*	17.5 % of PEF	*PosFVSl*	40 % FVC
**C7b**	**FV_Slope_test**	M_8	*Max_Slope*<br>*KSlopeTest*	16.75 % of PEF<br>2	*Min_Slope*<br>*TSlope*	1.1 L/s<br>0.1 s
**C7c**	**FVSlopeTest2**	M_8	*ThSlTest2*	20 % of PEF	*ThDerivTest*<br>*ThSlopeCalc*	0.010 L/s^2<br>0.5 L
**C7d**	**FVSlopeTestArea**	M_8 and M_9	*ThFVSlopeTArea*	0.01 L		

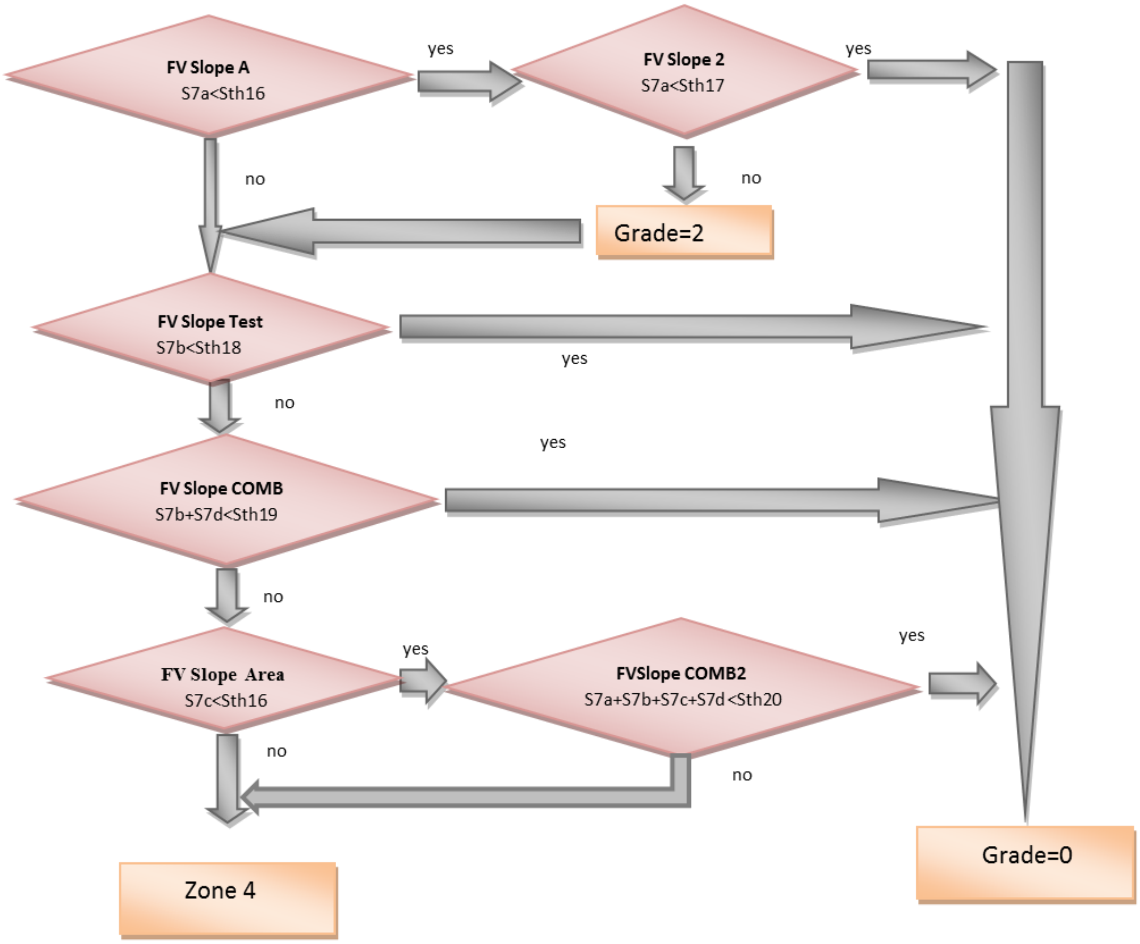

Figure S3 – Flowchart of the algorithm in Z3: in each decision box the curve scores (*Sn*) or the combination of scores are compared with the respective threshold (*Sthn*), the rectangular box are used to assign a grade to the curve or to go to the following zone.

Zone Z4 Analysis

This group of criteria has been developed to analyze the last two seconds of FV and VT

curves. The criteria ensure that the flow at the end of the curve is regular and free from fluctuations and are based on the first order derivative of FT curve in the zone Z4, and the difference between max and min volume in the last 1 s of exhalation. Table S4 lists the different threshold used for the comparison tests of the metrics.

A new metric M_{10} defines the *EOTV* as a function of *Tex*. The metric M_{11} defines a new threshold for *EOTV* (*EOTVth*) as a function of *Tex*. A new metric M_{12} is calculated in the FT curve as the slope at the curve end. Besides, in the end of VT curve a horizontal line of 1 s of length is generated and the area under and above this line is calculated (M_{13}).

Criteria C_8 is the traditional *BEV* criteria (*BEV*>0.15 L or *BEV* < 5% of *FVC*). Criteria C_9 is the traditional *EOTV* criteria (V < 0.025 L in $t \geq 1$ s). Criteria C_{10} is a combination of 5 criteria based on the interaction between traditional *EOTV* and *Tex* and the metrics M_{10} and M_{11}. C_{10a} detects if the *EOTV* and *Tex* both does not satisfy their traditional criteria. C_{10b} defines a new period to calculate *EOTV* if the *Tex* traditional criteria is satisfied. C_{10c} defines a new threshold for *EOTV* if the *Tex* traditional criterion is satisfied. C_{10d} uses threshold M_{10} to define the *EOTV*(*Tex*) and compare with the traditional threshold. C_{10e} defines a new threshold for *EOTV* (M_{11}) calculated as a function of *Tex*. Criterion C_{11}detects irregularity or oscillation at the end part of FT curve when the FT curve exceeds a threshold (*FT_endMx*) for subsequent values of F that occur after the point at which the first order derivative falls below a threshold (*FT_endMin*) of PEF. This value is combined with the value (M_{13}) of the area generated by a horizontal line of 1 s in the end of VT curve. Figure S4 depicts the flow-chart of the decision process in Z4.

TABLE S4

All the Z4 criteria associated with metrics and respective thresholds

n	CRITERION (C_n)	METRICS	MAIN THRESHOLD (th_{ni}) *i=1*	VALUE	SECONDARY THRESHOLD (th_{ni}) *i={2,3}*	VALUE
**C8**	**BEV**	BEV	*BEVth*	0.150 L		
**C9**	**EOTVtrad**	EOTV	*EOTVth*	0.025 L		
**C10a**	**EOTV_Tex**	EOTV and T_{ex}	*TmaxEx*	6 s		
**C10b**	**EOTV_Tex_05s**	EOTV and T_{ex}	*EOTVth*	0.025 L	*TEotv*	0.5 s
**C10c**	**EOTV_Tex_Th01**	EOTV and T_{ex}	*EOTVth2*	0.050 L		
**C10d**	**EOTV(Texp)**	M_{10}	*EOTVth*	0.025 L		
**C10e**	**EOTV_Tex_ThVar**	M_{11}	*EOTVthDyn*	$EOTVth(Tex) = EOTVth\ \frac{Tex}{6}$		
**C11**	**FT_end**	M_{12} and M_{13}	*FT_endMx*<br>*AuVTh*	5 % of PEF<br>0.025 L	*FT_endMin*	0.00001 L/s

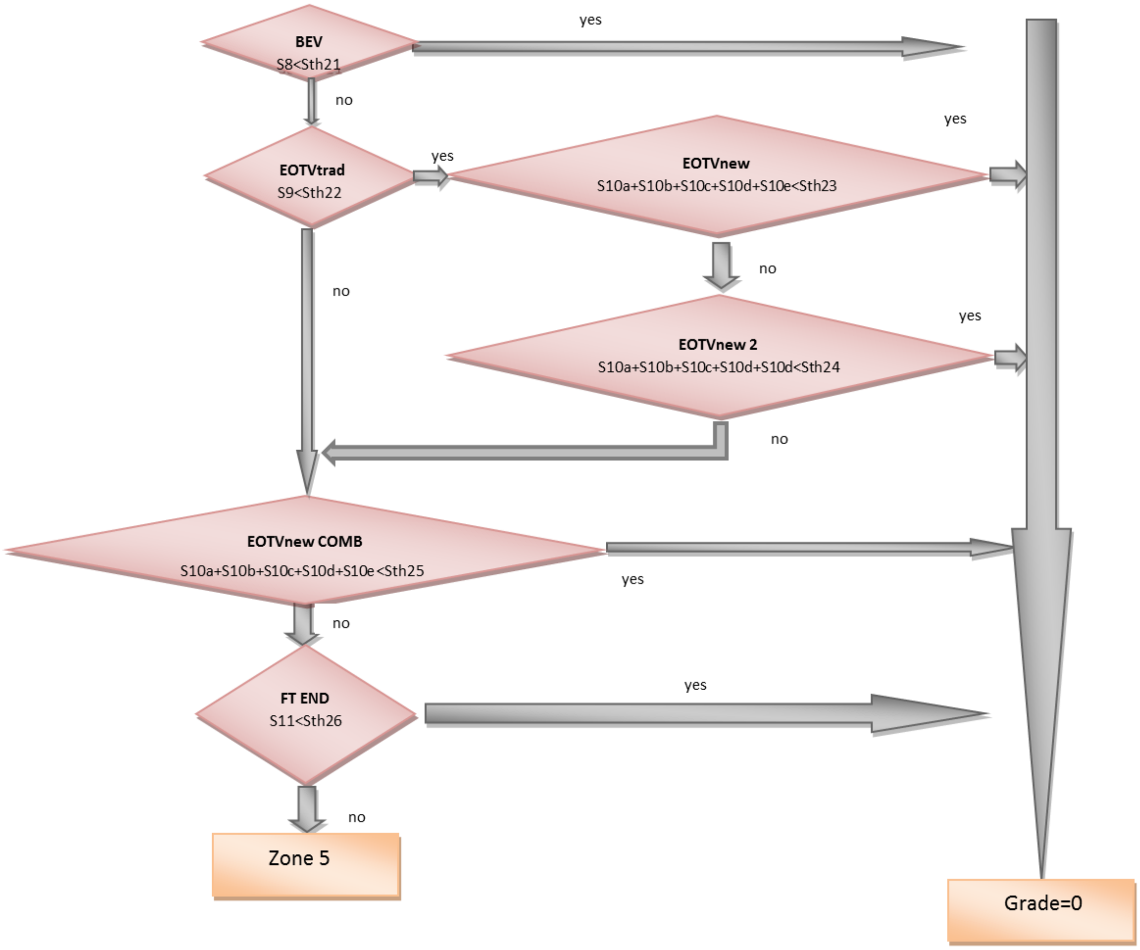

Figure S4 – Flowchart of the algorithm in Z4: in each decision box the curve scores (*Sn*) or the combination of scores are compared with the respective threshold (*Sthn*), the rectangular box are used to assign a grade to the curve or to go to the following zone.

Zone Z5 Analysis

This group of criteria has been developed to analyze the morphology of the entire FV curve. The criteria ensure that there only exists one local maximum (the *PEF* point) and they are based on the derivative of the FV curve. Table S5 lists the different threshold used for the comparison tests of the metrics.

The metric M_{14} is defined as the maximum of the difference between all local minimum and consecutive local maximum on the FV curve detected by analyze the sign of the first order derivative of the FV curve.

The criterion C_{12a} detects a situation of multiple peaks when the M_{14} exceeds a certain percentage threshold (*PVFVTh1*) of the *PEF* that typically occurs when the subject coughs. The criterion C_{12b} detects a situation of multiple peaks for values of *V* adjacent to *FEV1* when the height of secondary local maxima exceeds a threshold function of the distance to *FEV1* (*PVFVThDyn*). Figure S5 depicts the flow-chart of the decision process in Z5.

TABLE S5

All the Z5 criteria associated with metrics and respective thresholds

N	CRITERION (C_n)	METRICS	MAIN THRESHOLD (th_{ni}) *i=1*	VALUE
C12a	Peak_Valley_Single	M_{14}	*PVFVTh1*	5 % of PEF
C12b	Peak_Valley_Combined	M_{14}	*PVFVThDyn= dFEV1 PVFVTh1*	*PVFVTh1*= 2.5 % of PEF<br>*dFEV1*= $100 * \frac{\lvert F_{pv}-F_{FEV1}\rvert}{FVC}$

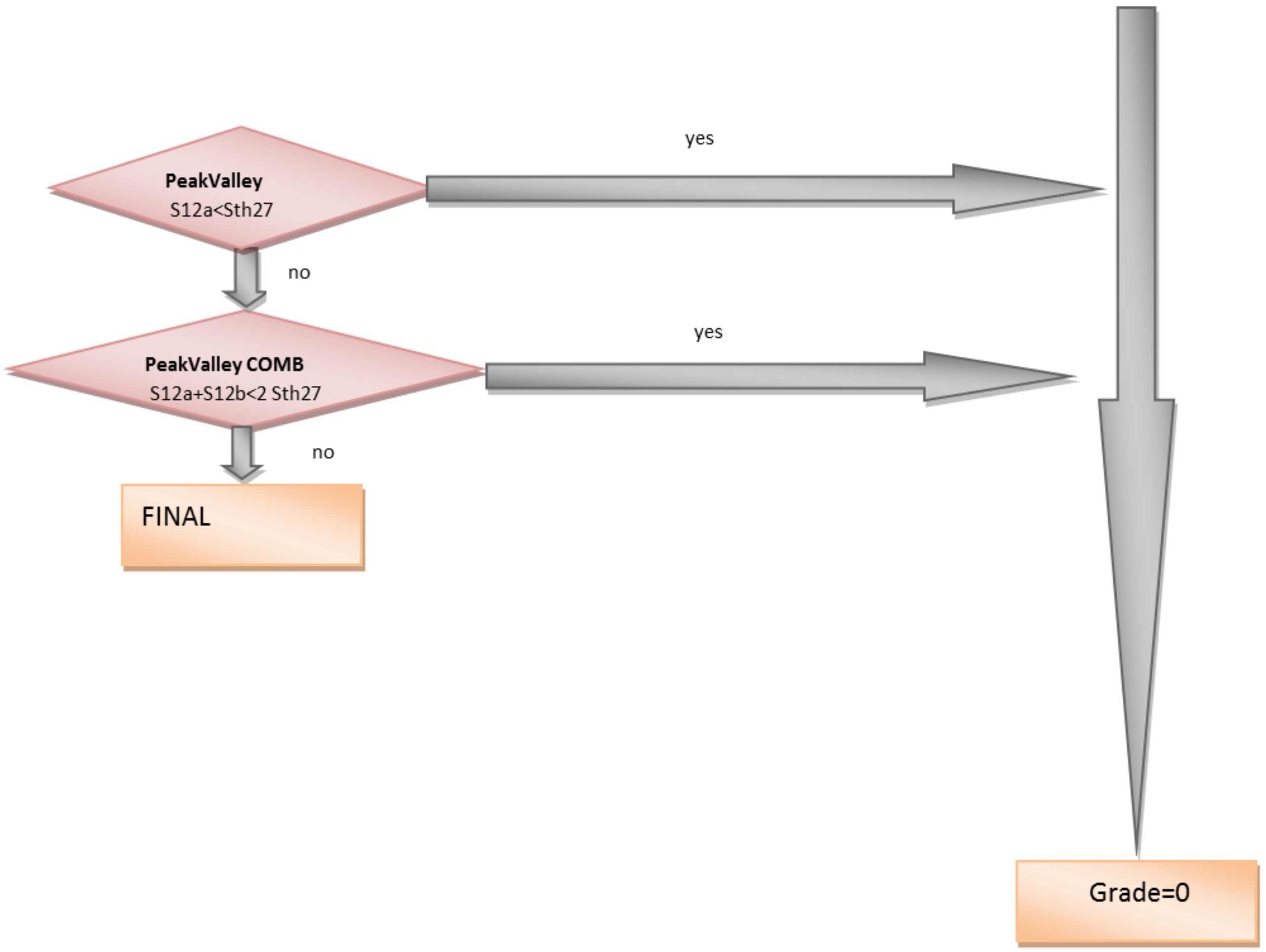

Figure S5 – Flowchart of the algorithm in Z5: in each decision box the curve scores (*Sn*) or the combination of scores are compared with the respective threshold (*Sthn*), the rectangular box are used to assign a grade to the curve or to go to the following zone.

The Final Control

The final control (Figure S6) is based on S_{13} (TOT SCORE) that is calculated as the sum of all the S_n of all the criteria (n): $S_{13} = \sum_n S_n$.

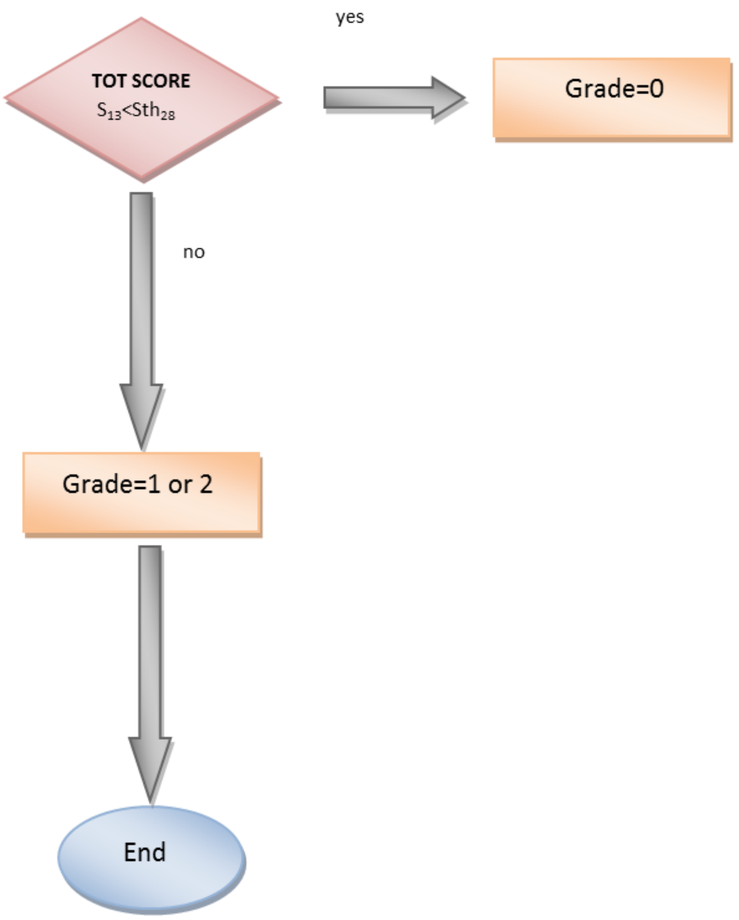

(f)

Figure S6 – Flowchart of the algorithm of the Final control.

Table S6 contains a list of all the score thresholds associated with the criteria.

TABLE S6

All the score threshold values of each block

CODE	CRITERIA	BLOCK	SCORE THRESHOLD (STh_n)
**Sth1**	C_1 and C_{2b}	Diff_single/Area Diff2	50
**Sth 2**	C_{2a}	AreaDiff	100
**Sth 3**	$C_{1,}$ C_{2a} and C_{2b}	Diff Combined	250
**Sth 4**	C_3 and C_4	PEF Time Up/Down	50
**Sth 5**	C_3 and C_4	PEF Time UP/Down 2	75
**Sth 6**	C_{5a}	PEF Cut	50
**Sth 7**	C_{5a}	PEF Cut 2	75
**Sth 8**	C_{5b}	PEF Cut FEV1	50
**Sth 9**	C_{5a} and C_{5b}	PEF Peak COMB 2	75
**Sth 10**	C_{5a} and C_{5b}	PEF Peak COMB	100
**Sth 11**	C_{5c}	PEF Double	50
**Sth 12**	C_{5a}, C_{5b} and C_{5c}	PEF Double COMB	150
**Sth 13**	C_{5a}, C_{5b} and C_{5c}	PEF Double COMB 2	125
**Sth 14**	C_6	PEF Slow	50
**Sth 15**	C_6	PEF Slow 2	75
**Sth 16**	C_{7a} and C_{7c}	FV Slope / FV Slope	40
**Sth 17**	C_{7a}	FV Slope 2	75
**Sth 18**	C_{7b}	FVSlope Test	50
**Sth 19**	C_{7b} and C_{7d}	FVSlope COMB	175
**Sth 20**	C_{7a}, C_{7b}, C_{7c} and C_{7d}	FVSlope_COMB 2	160
**Sth 21**	C_8	BEV	100
**Sth 22**	C_9	EotvTrad	100
**Sth 23**	C_{10a}, C_{10b}, C_{10c}, C_{10d}, C_{10e}	EOTVnew	350
**Sth 24**	C_{10a}, C_{10b}, C_{10c}, C_{10d}, C_{10e}	EOTVnew 2	400
**Sth 25**	C_{10a}, C_{10b}, C_{10c}, C_{10d}, C_{10e}	EOTV new COMB	360
**Sth 26**	C_{11}	FTend	40
**Sth 27**	C_{12a} and C_{12b}	Peak_Valley/PeakValley	50
**Sth 28**	C_1-C_{12b}	TOT SCORE	1350
